# Signaling mechanisms that regulate ex vivo survival of human acute myeloid leukemia initiating cells

**DOI:** 10.1038/s41408-017-0003-1

**Published:** 2017-11-30

**Authors:** Dheeraj Bhavanasi, Kwun Wah Wen, Xiaolei Liu, Francois Vergez, Gwenn Danet-Desnoyers, Martin Carroll, Jian Huang, Peter S Klein

**Affiliations:** 1 Department of Medicine (Hematology-Oncology), University of Pennsylvania Perelman School of Medicine, Philadelphia, PA USA; 2 Institute of Hematology and Blood Diseases, Chinese Academy of Medical Sciences, Tianjin, China; 3 Department of Pathology and Laboratory Medicine, Temple University School of Medicine, Philadelphia, PA USA; 4 Present Address: Department of Pathology and Laboratory Medicine, University of California, San Francisco, CA USA

Acute myeloid leukemia (AML) is an aggressive malignancy of the hematopoietic system that arises through clonal expansion of myeloid precursor cells that have arrested at an early stage of differentiation and ultimately causes death in over 50% of patients. Despite the aggressive growth characteristics of AML in vivo, AML blasts are difficult to culture once removed from the patient, suggesting that AML cells depend on signals from the microenvironment^1,2^. AML initiating cells (LICs), defined functionally as cells capable of initiating AML in immunocompromised mice^3^, are also challenging to maintain in culture, presenting a major obstacle to developing new therapies for AML that target the LIC. Co-culture with stromal cells^4–6^ can maintain and expand LICs; however these culture systems use multiple cytokines that may promote differentiation and loss of LICs ex vivo. Small molecules such as SR1 (an aryl-hydrocarbon receptor antagonist) and UM729 support AML viability without stromal co-culture, but also require multiple cytokines^1^. In this work, we demonstrate an alternative approach that enhances the viability of primary AML cells and supports ex vivo maintenance of LICs in cytokine-free medium. We show that inhibition of GSK-3, which activates Wnt signaling, markedly improves the viability of primary human AML cells in cytokine-free medium and further that combined inhibition of GSK-3 and the nutrient sensor mTORC1 maintains LICs capable of engrafting immunocompromised mice, identifying a signaling network that regulates the viability of LICs.

As GSK-3 inhibition expands hematopoietic stem and progenitor cells^7^ and *Gsk3* knockout causes a myeloproliferative neoplasm suggestive of AML in mice^8^, we tested whether direct inhibition of GSK-3 would enhance the viability of primary AML cells ex vivo. AML cells in culture do not proliferate and typically die over a few days. Cells from AML patients were cultured in defined medium without cytokines in the presence of the GSK-3 inhibitor CHIR99021 (Chiron) or vehicle. After 5 days, cells cultured with vehicle alone were small with fragmented nuclei whereas cells cultured with Chiron were uniformly larger with intact, round nuclei (Fig. 1a). The fraction of viable cells upon GSK-3 inhibition increased in a dose-dependent manner over 10 days (Fig. 1b, Supplementary Fig. 1a), with a maximal effect at 1–3 µM Chiron. The mechanistically distinct GSK-3 inhibitor, lithium chloride, also increased the fraction of viable cells compared to control at concentrations similar to the established IC_50_ for lithium inhibition of GSK-3 (Figs. 1c, Supplementary Fig. 1b). To address potential off-target effects with these inhibitors, we knocked down *GSK3* with short interfering RNAs (siRNAs). Knockdown (30% of control for *GSK3A* and *GSK3B*, Supplementary Fig. 1c, d) of either *GSK3A* or *GSK3B* alone had no effect on viability, but combined knockdown significantly increased the viable fraction relative to control siRNA, similar to chemical GSK-3 inhibitors (Gsk3i) and consistent with redundant functions for *GSK3A* and *GSK3B* ^9^ (Supplementary Fig. 1e). Thus GSK-3 inhibition improves the viability of AML cells ex vivo.

**Fig. 1:**
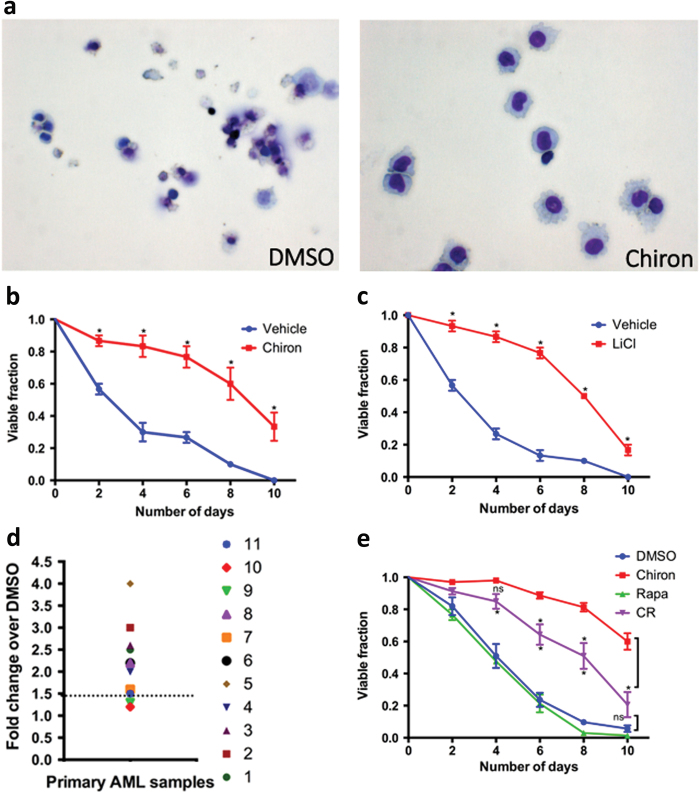
GSK3i increases viability of primary human AML cells **a** 5 × 10^6^ primary human AML cells were cultured in the presence of DMSO or Chiron (3 μM) for 5 days and cytospin was performed. **b** AML cells were cultured in the presence of Vehicle, Chiron, or **c** LiCl (5 mM) for 10 days under cytokine-free conditions. The viable fraction was determined every other day by trypan blue staining. The plots represent viable fraction vs number of days (*n* = 3). **d** Eleven AML patient samples were cultured in the presence of DMSO or Chiron and the viable fraction was determined by trypan blue staining. Data are represented as fold change in % viable cells relative to vehicle (DMSO) control. (Cells from each patient were tested in culture at least two times and in most cases >3 times). **e** Human AML cells were cultured in the presence of DMSO, Chiron, Rapamycin (10 nM), or Chiron + Rapamycin (CR) and the viable fraction was measured every other day for 10 days. The viable fraction was determined by trypan blue staining and represented as viable fraction vs number of days (*n* = 3). * indicates *p* < 0.05, ns = not significant

To test the generality of the response to GSK-3 inhibition, primary AML blasts with distinct genetic and pathologic characteristics from eleven patients (Supplementary Table 1) were examined. Gsk3i enhanced the viability of primary AML cells ≥1.5 fold compared to control in 9 of 11 patients tested (Fig. 1d, Supplementary 2a). Enhanced viability was independent of FAB/WHO (French-American-British/World Health Organization) classification or the mutational status of Flt3 or NPM1, indicating that reduced viability due to GSK-3 activation *ex vivo* could be a general feature of primary AML cells from a wide range of patients.

GSK-3 antagonizes Wnt signaling by phosphorylating β-catenin and targeting it for degradation. Gsk3i mimics Wnt signaling by preventing β-catenin phosphorylation, leading to protein stabilization, which then activates Wnt target genes. Wnt inhibition of GSK-3 also activates mTORC1 in diverse cell types including hematopoietic cells^10–12^. To address the role of mTORC1 signaling in maintaining AML cell viability, we cultured cells in the presence of DMSO, Chiron, rapamycin, or Chiron + rapamycin (CR) and measured cell viability; CR reduced viability significantly compared to Chiron alone (Fig. 1e), indicating that the enhanced viability ex vivo requires mTORC1 activation. Cytospin preparations of CR treated AML cells confirmed the presence of cells with fragmented nuclei (Supplementary Fig. 2b), similar to but less severe than vehicle alone. In contrast, siRNA knockdown of β-catenin had no effect on AML cell viability (Supplementary Fig. 2c, d). These observations support a role for mTORC1 downstream of GSK-3 in maintaining viability of AML cells ex vivo.

As AML stem and progenitor cells are typically lost during culture, we performed colony formation assays to determine their functional potential under our culture conditions. Chiron treated cells contained ~5-fold more colony forming cells (CFCs) than control cells (Fig. 2a), consistent with enhanced viability of a progenitor population in the Gsk3i cultures. AML cells cultured in CR contained fewer CFCs than those cultured in Chiron, but still 2-fold higher than the control cells. To determine whether LICs were maintained under these culture conditions, we transplanted cultured AML cells into NSG mice (Supplementary Fig. 3). Engraftment of human CD45^+^CD33^+^ cells was measured 10 weeks after transplantation. Uncultured AML cells engrafted with an approximate LIC frequency of 1 in 63,827 (95% CI: 22,019–185, 018) (Fig. 2b) whereas cells from the same patient cultured for 4 days with vehicle, DMSO or with Chiron failed to engraft (Fig. 2c). Cells cultured in CR engrafted in all surviving mice, demonstrating that CR maintains LICs ex vivo in the absence of cytokines or support cells. To extend this to additional patient samples, we tested the effect of CR culture on the maintenance of LICs from three other AML patients. Primary AML cells from two additional patients engrafted when cultured in CR but not DMSO (Fig. 2d).

**Fig. 2:**
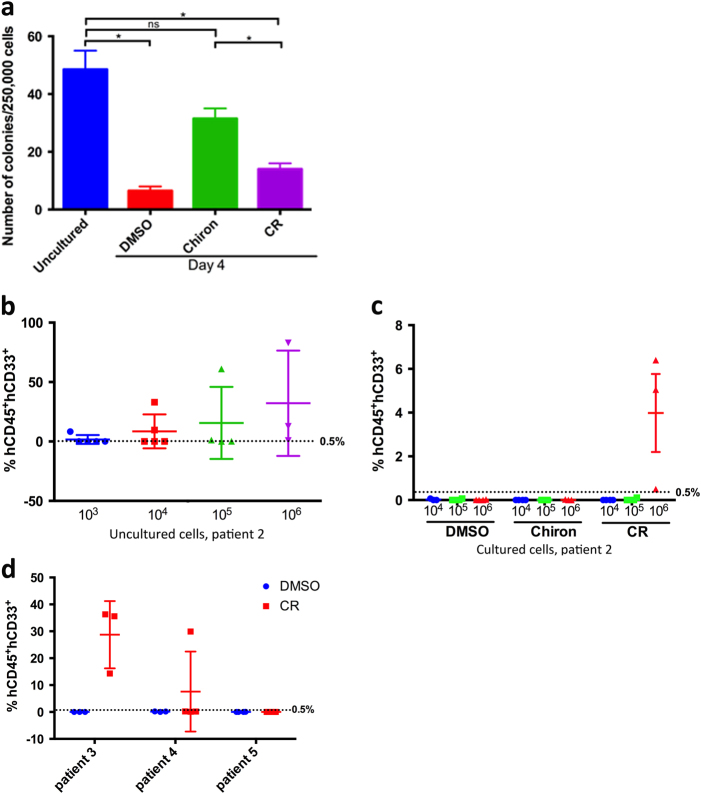
Combined inhibition of GSK-3 and mTORC1 maintains leukemia initiating cells ex vivo **a** For CFC assays, AML cells were cultured for 4 days with DMSO, Chiron, or CR and then placed in methylcellulose for colony formation assay. An equal number of uncultured cells were placed directly into methylcellulose for colony formation assays. Total number of colonies obtained after 14 days is shown for each condition. **b** Scatter plots showing number of transplanted mice and mice positive for engraftment (>0.5% hCD45^+^hCD33^+^) after 10 weeks of transplantation for different doses of uncultured cells and **c** cells cultured in DMSO, Chiron, or CR for cells derived from same patient. Black dotted line represents 0.5% hCD45^+^hCD33^+^. **d** Scatter plot showing number of transplanted mice and mice positive for engraftment after 10 weeks of transplantation for cells cultured in DMSO or CR for three other patients. Black dotted line represents 0.5% engraftment. * indicates *p* < 0.05, ns = not significant

Data from this study and our previous work with nonmalignant HSCs suggest that inhibition of GSK-3 and mTORC1 is a general mechanism to maintain self-renewing hematopoietic cells from healthy donors^10^ or AML patients. β-catenin is essential for long-term HSC self-renewal in response to Gsk3i^10^, but the parallel activation of mTORC1 drives HSCs to proliferate and enter more differentiated lineages, leading to HSC exhaustion, as also observed with *Pten* knockout in hematopoietic cells^13,14^. Similarly, activation of mTORC1 by GSK3i prevents AML cells from engrafting whereas LICs are maintained with GSK3i and parallel inhibition of mTORC1. Consistent with these observations, knockout of *Gsk3a* and *Gsk3b* in mouse bone marrow causes a severe myeloproliferative neoplasm with increased blasts and features of AML^8^. The mechanism downstream of GSK-3 again appeared to be through enhanced Wnt/β-catenin signaling, although other effectors may contribute. Taken together, these findings support a model in which AML cells in vivo are supported by signals from the microenvironment that inhibit GSK-3 and that activation of GSK-3 could serve as a strategy to treat AML.

In conclusion, we show that the signaling molecules GSK-3 and mTORC1 regulate the viability of primary human acute myeloid LICs. Combined inhibition of GSK-3 and mTORC1 maintains LICs under cytokine-free conditions, suggesting a novel approach to study signaling in primary AML cells, screen for new therapies for AML, and test the molecular response to targeted AML therapies in a patient-specific manner.

## Methods

### Primary human AML cell culture

De-identified cells from patients with AML were obtained from the Stem Cell and Xenograft Core (SCXC) at the University of Pennsylvania. In total 5 × 10^6^ cells/ml were cultured in cytokine-free medium in the presence of DMSO or PBS (vehicle control) or the indicated concentrations of Chiron, LiCl, and/or Rapamycin for 4–10 days. Viability was measured by trypan blue dye exclusion every other day for time course experiments and on day 4 for dose response experiments. Viable fraction was calculated as the number of live cells/(live cells+dead cells).

### Xenotransplantation

Transplantations were performed through the SCXC. Non-obese diabetic severe combined immunodeficient IL-2Rγ null (NSG) mice were conditioned with busulphan or irradiation 24 h before intravenous or intrafemoral injection of cells. 10^4^, 10^5^, and 10^6^ AML cells were injected prior to culture and an equivalent volume of the remaining culture was injected after 4 days into 10-12 week NSG mice. After 10 weeks of transplantation, engraftment was measured by flow cytometry for hCD45^+^hCD33^+^ cells. Engraftment of 0.5% was considered as positive.

Detailed description of other methods is provided in supplement.

## Electronic supplementary material

Supplemental Data and Methods
