# Supplementary material for: Signaling mechanisms that regulate ex vivo survival of human acute myeloid leukemia initiating cells
Source: Blood Cancer J. 2017 Nov 30;7(12):636. doi: 10.1038/s41408-017-0003-1 (PMC5802493; doi:10.1038/s41408-017-0003-1)
Supplement: Supplementary file 1 — Supplemental Data and Methods [file 41408_2017_3_MOESM1_ESM.pdf]

METHODS:

Primary human AML cell culture:

AML cells were primarily blasts isolated by apheresis and stored in liquid nitrogen. The cells were thawed and washed with RPMI 1640 medium+2% heat inactivated serum and pre-incubated for 2 hours in fresh RPMI medium. Cells were then passed through a 40μm cell strainer; live cells were counted by trypan blue dye exclusion and subsequently $5X10^6$ cells were cultured in cytokine-free medium with DMSO (vehicle control), 3 μM Chiron 99021, or Chiron + 10 nM Rapamycin (CR).

Flow cytometry:

Bone marrow aspirates or flushed bone marrow samples from the transplanted NSG mice were incubated with PE-hCD45, FITC-hCD33 (Biolegened) for 20 minutes in 2.5 % FBS-PBS. Non-viable cells were excluded using the viability dye DAPI (1μg/ml). Measurements were performed on a FACSCanto (Becton Dickinson) and data were analyzed with FlowJo software.

Cytospin preparation:

AML cells were cultured in DMSO, Chiron, or CR for 5 days: re-suspended at 20,000 cells/ml in PBS, and spun in a cyto centrifuge at 1000 rpm, medium speed for 5 minutes onto superfrost microscopic slides with a cytofunnel. The slides were fixed and stained with Hema3 staining solution (Fischer Scientific), and photographed.

siRNA transfection:

$1X10^6$ primary AML cells were nucleofected with 1μM non-targeting siRNA or siRNAs targeting either *GSK3A* or *GSK3B* or both or β-catenin obtained from Dharmacon and viable fraction was determined as mentioned above on days 2, 4 and 6.

Colony forming cell assay:

250,000 AML cells cultured for 4 days in the presence of DMSO, 3 μM Chiron, or CR (and an equal number of uncultured cells) were transferred to methylcellulose (HSC005 from R&D

Technologies) with cytokines. After 14 days, colonies were counted with STEMgrid (STEMCELL Technologies), under a microscope.

Real time PCR:

Primary AML cells nucleofected with siRNA were centrifuged and RNA was isolated using the RNeasy Plus Micro kit (Qiagen) as indicated by the manufacturer. 50 ng total RNA per sample was used for first-strand cDNA synthesis using SuperScript III reverse transcriptase (Invitrogen) as indicated by the manufacturer. Relative gene expression was quantified on a 7900HT Fast Real-Time PCR System (Applied Biosystems) using Power SYBR Green PCR Master Mix (Applied Biosystems). Expression levels of genes of interest were normalized to h*GAPDH* expression.

Western Blotting:

Primary human AML patient cells were transfected with non-targeting siRNA and siRNA targeting β-catenin and after 24 hrs, cells were lysed by repeated freeze-thawing in lysis buffer containing Triton X-100 and EDTA. Proteins were separated by 10% SDS–polyacrylamide gel electrophoresis and immunoblotting was performed with antibodies for ß-catenin (1:1000) (Cell Signaling Technology) and total actin (1:5000) (Cell Signaling Technology) and immunoreactivity was detected by chemiluminescence using a Fuji Film Image analyzer.

Statistical Analysis:

The results were quantified and expressed as means ± SEM. Statistical significance was tested by Student's t test. P value < 0.05 was considered significant and represented as *. ns = not significant.

SUPPLEMENTAL FIGURE LEGENDS:

Figure S1: GSK3i or GSK-3 knock down increases the viable fraction of primary human AML cells.

(a) 5 X 10^6 AML cells were cultured in RPMI 1640 medium with 2% heat inactivated serum in the presence of DMSO or indicated concentrations of Chiron or (b) LiCl. The plot represents viable fraction at different doses of Chiron or LiCl (n=3) (c) 1 X 10^6 AML cells were nucleofected with 1 μM non-targeting siRNA or siRNAs targeting *GSK3A, GSK3B,* or both. At 24 hrs post nucleofection, expression of *GSK3A* or (d) *GSK3B* was determined by quantitative real time PCR. *GSK3A* and *GSK3B* expression is plotted as a percentage of control siRNA. (e) The viable fraction was determined on days 2, 4 and 6 (n=3). * indicates $p<0.05$, ns = not significant.

Figure S2: Inhibition of mTORC1 but not β-catenin affects AML cell survival.

(a) AML cells (5 X 10^6 cells) from 11 patients were cultured in the presence of DMSO or Chiron and the viable fraction was measured after 4 days by trypan blue dye exclusion. (b) Cytospin images of primary AML cells cultured for 5 days with DMSO, Chiron, or CR. (c) 1 X 10^6 human AML patient cells were nucleofected with 1 μM non-targeting siRNA or siRNA targeting β-catenin and cultured in the presence of DMSO or 3 μM Chiron and the viable fraction was measured on days 2, 4 and 6. (d) 24 hrs post nucleofection, cells were harvested for western blot for β-catenin. Actin was used as a loading control. ns = not significant.

Figure S3: Xenotransplantation of primary human AML cells.

(a) Experimental design for xenograft: 10^4, 10^5 and 10^6 AML cells were transplanted immediately (uncultured) or cultured for 4 days and then transplanted into NSG mice. After 10 weeks of transplantation, engraftment was measured by flow cytometry for $hCD45^+hCD33^+$ cells. Engraftment of ≥ 0.5% was considered positive for engraftment.

Table S1: AML patient characteristics

Abbreviations: FAB/WHO: French-American-British/World Health Organization, FLT3: Fms-related tyrosine kinase 3, ITD: internal tandem duplication, NPM: Nucleophosmin

Patient ID	FAB/WHO subtype	Age	Sex	FLT3	NPM	Cytogenetics
1	M4 [myelomonocytic]	52	M	ITD	Not available	Normal
2	AML-MLD with prior MDS	68	F	Wildtype	Not available	46,XX[20]
3	M4 [myelomonocytic]	59	F	ITD	Not available	Normal
4	Not available	39	M	ITD	Mutant	Normal
5	M5 (combining both M5a and M5b)	46	F	ITD	Mutant	Normal
6	M1 [without maturation]	52	M	Wildtype	Wildtype	46,XY,del(2)(p13p?23),t(4;13)(q31;q34),add(4)(q?25),del(6)(q13q25),t(9;22)(q34;q11.2),del(10)(q24),add(16)(q24)[20]
7	AML-MLD no prior MDS or MPN	64	M	ITD	Wildtype	47,XY,+8[2]/46,XY,t(3;17)(q21;q25)[2]/46,XY[16]
8	AML-MLD with prior MPN	66	M	Wildtype	Wildtype	46,XY,del(20)(q11.2q13.1)[20]
9	Not available	79	M	Not available	Not available	Normal
10	Not available	54	F	ITD	Mutant	Normal
11	M4 [myelomonocytic]	52	M	ITD	Not available	46,XY,add(6)(p21),del(8)(p21),add(12)(q24.1)[13]/46,XY,del(1)(q32),del(7)(q22q32),der(6;12)(q10;p10),add(22)(q?11.2),+mar[3]/45,XY,t(1;2)(p?22;q11.2),-21[1]/46,XY[3]

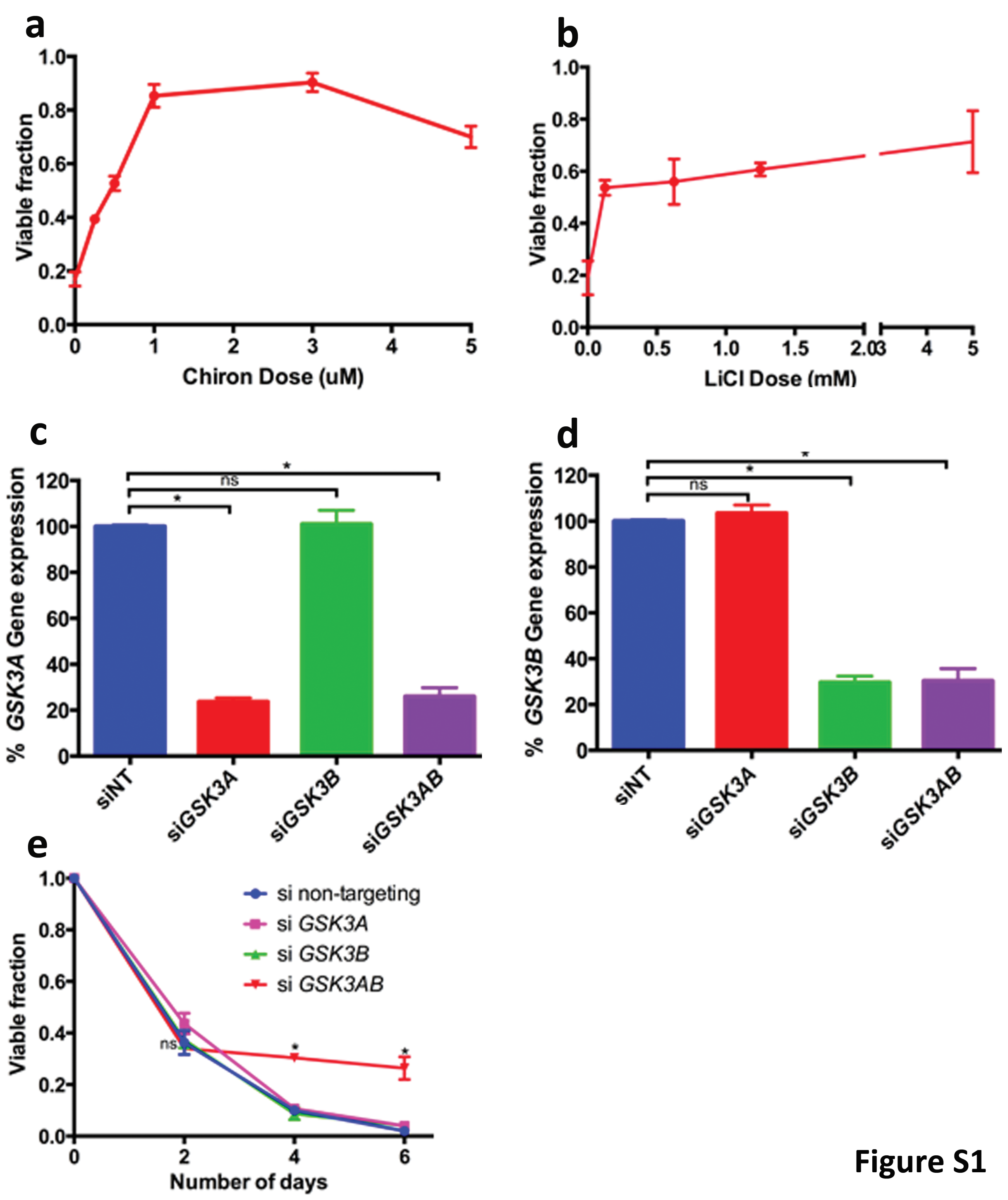
a
1.0
0.8
0.6
0.4
0.2
0.0
Viable fraction
0
1
2
3
4
5
Chiron Dose (uM)
b
1.0
0.8
0.6
0.4
0.2
0.0
Viable fraction
0.0
0.5
1.0
1.5
2.03
4
5
LiCl Dose (mM)
c
% GSK3A Gene expression
120
100
80
60
40
20
0
*
ns
*
siNT
siGSK3A
siGSK3B
siGSK3AB
d
% GSK3B Gene expression
120
100
80
60
40
20
0
*
*
ns
siNT
siGSK3A
siGSK3B
siGSK3AB
e
1.0
0.8
0.6
0.4
0.2
0.0
Viable fraction
si non-targeting
si GSK3A
si GSK3B
si GSK3AB
ns
*
*
0
2
4
6
Number of days

Figure S1

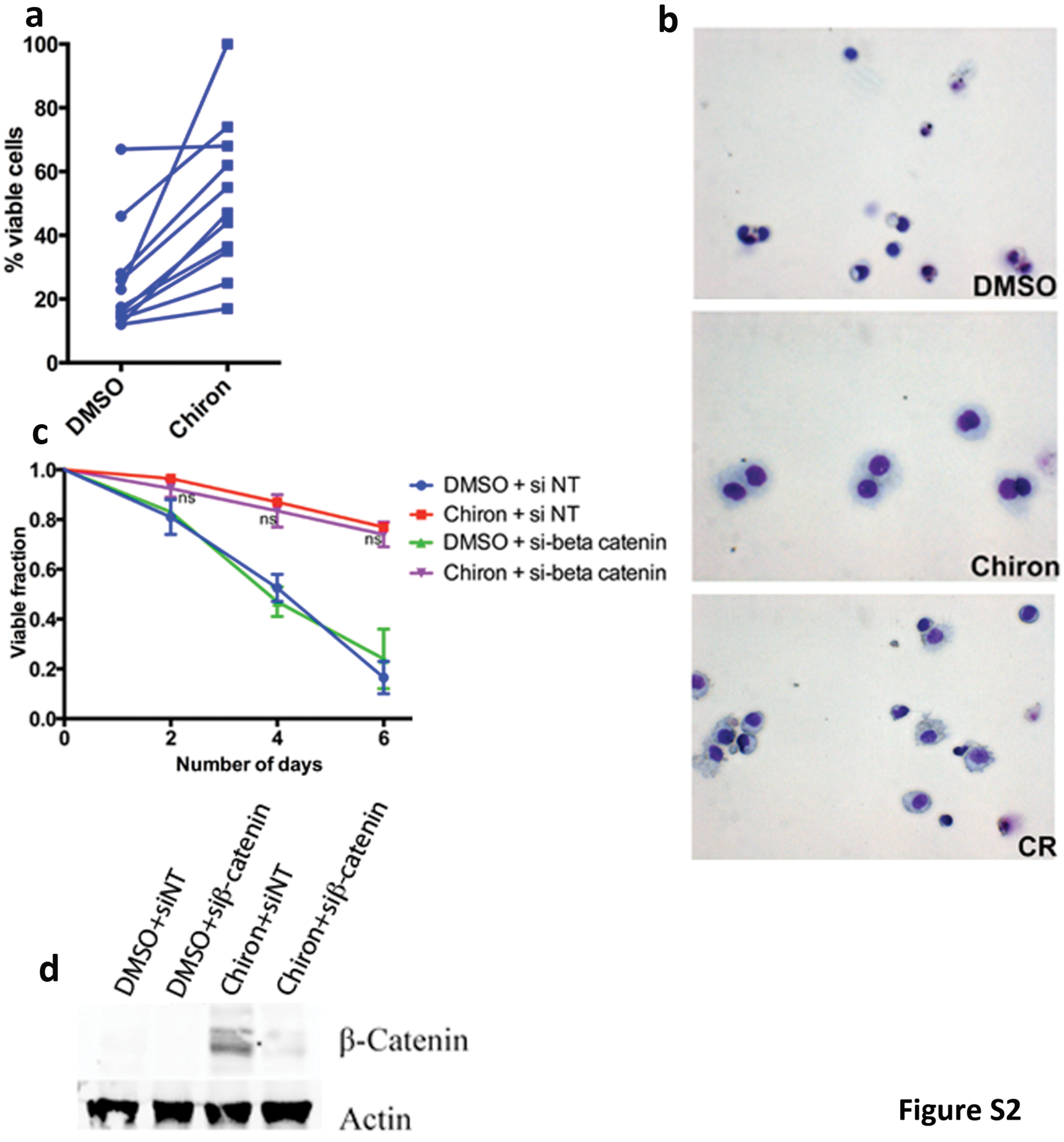
a
100
80
60
40
20
0
% viable cells
DMSO
Chiron
b
DMSO
Chiron
CR
c
1.0
0.8
0.6
0.4
0.2
0.0
Viable fraction
0
2
4
6
Number of days
ns
DMSO + si NT
Chiron + si NT
DMSO + si-beta catenin
Chiron + si-beta catenin
d
DMSO+siNT
DMSO+siβ-catenin
Chiron+siNT
Chiron+siβ-catenin
β-Catenin
Actin

Figure S2

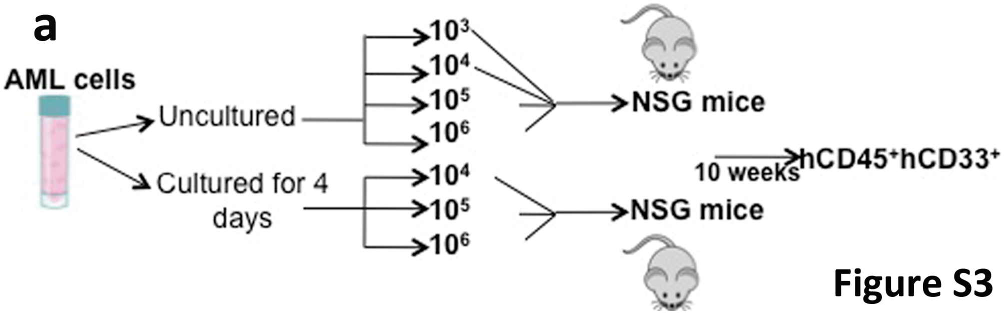
a
AML cells
Uncultured
Cultured for 4 days
10³
10⁴
10⁵
10⁶
10⁴
10⁵
10⁶
NSG mice
NSG mice
10 weeks
hCD45⁺hCD33⁺

Figure S3
